# Posttraumatic checkrein deformity following isolated Lauge–Hansen pronation external rotation stage IV malleolar fracture—a case report and literature review

**DOI:** 10.3389/fsurg.2023.887611

**Published:** 2023-02-09

**Authors:** Wen-tao Chen, Zhen-yu Liu, Bao-jun Wang

**Affiliations:** Department of Orthopaedics, Beijing Friendship Hospital, Capital Medical University, Beijing, China

**Keywords:** checkrein deformity, malleolar fracture, flexor hallucis longus, adhesion, Lauge–Hansen

## Abstract

The checkrein deformity is characterized by flexion contracture of the interphalangeal joint and extension contracture of the metatarsophalangeal joint. It is a rare condition occurring after lower extremity trauma, especially a malleolar fracture. Little is known about the possible cause and therapeutic strategy. This unique case presents a 20-year-old male patient with a diagnosis of the checkrein deformity secondary to open reduction and internal fixation of a Lauge–Hansen pronation external rotation stage IV malleolar fracture. After performing a detailed physical examination, radiographic evaluation, and ultrasonography, open exploration was performed to remove the hardware and correct the deformity with sole tenolysis of the flexor hallucis longus (FHL). In the 4-month follow-up, no recurrence of the checkrein deformity was observed. This deformity was caused by FHL adhesion. Interosseous membrane injury and fibular fracture together with local hematomas increases the risk of FHL adhesion. Open exploration and tenolysis of the FHL are feasible options to correct the checkrein deformity.

## Introduction

The checkrein deformity of the hallux is characterized by flexion contracture of the interphalangeal (IP) joint and extension contracture of the metatarsophalangeal (MP) joint (1). It is usually caused by the pathogenesis of flexor hallucis longus (FHL). A malleolar fracture is a common lower extremity trauma caused by rotational force. Although associated with soft tissue problems, open reduction and internal fixation (ORIF) might decrease posttraumatic arthritis. The complications of a malleolar fracture usually include malunion, nonunion, and infection (2). To our knowledge, this rare complication, checkrein deformity, has rarely been reported following Lauge–Hansen pronation external rotation (PER) of a stage IV malleolar fracture. Little is known about the diagnosis and therapeutic strategy. We present a unique case of checkrein deformity of the hallux and second toe following a Lauge–Hansen PER stage IV malleolar fracture. The clinical presentation, radiographic evaluation, ultrasonography, and surgical intervention were introduced to highlight this rare complication and therapeutic strategy.

## Case report

**Figure 1:**
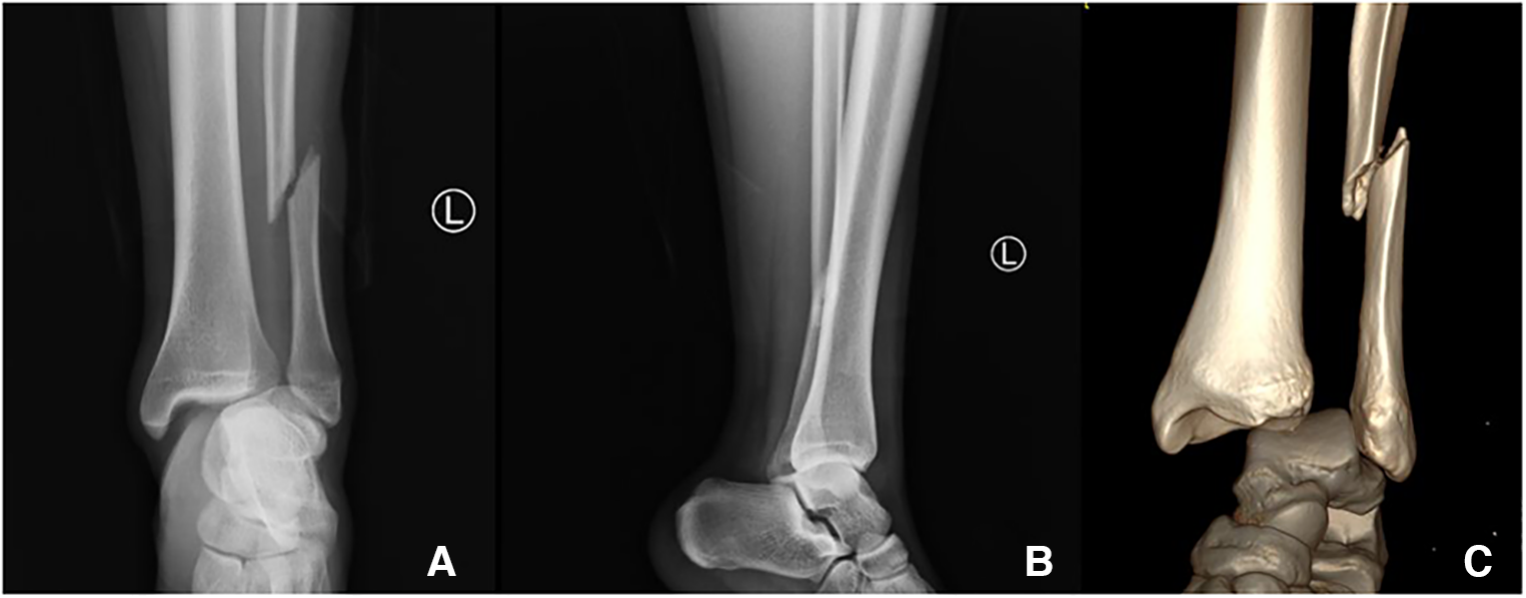
X-ray (**A**, anteroposterior view and **B**, lateral view) and 3D reconstruction of a CT scan (**C**) of a primary left ankle injury. Radiographic evaluation confirmed the diagnosis of a Lauge–Hansen PER stage IV malleolar fracture.

**Figure 2:**
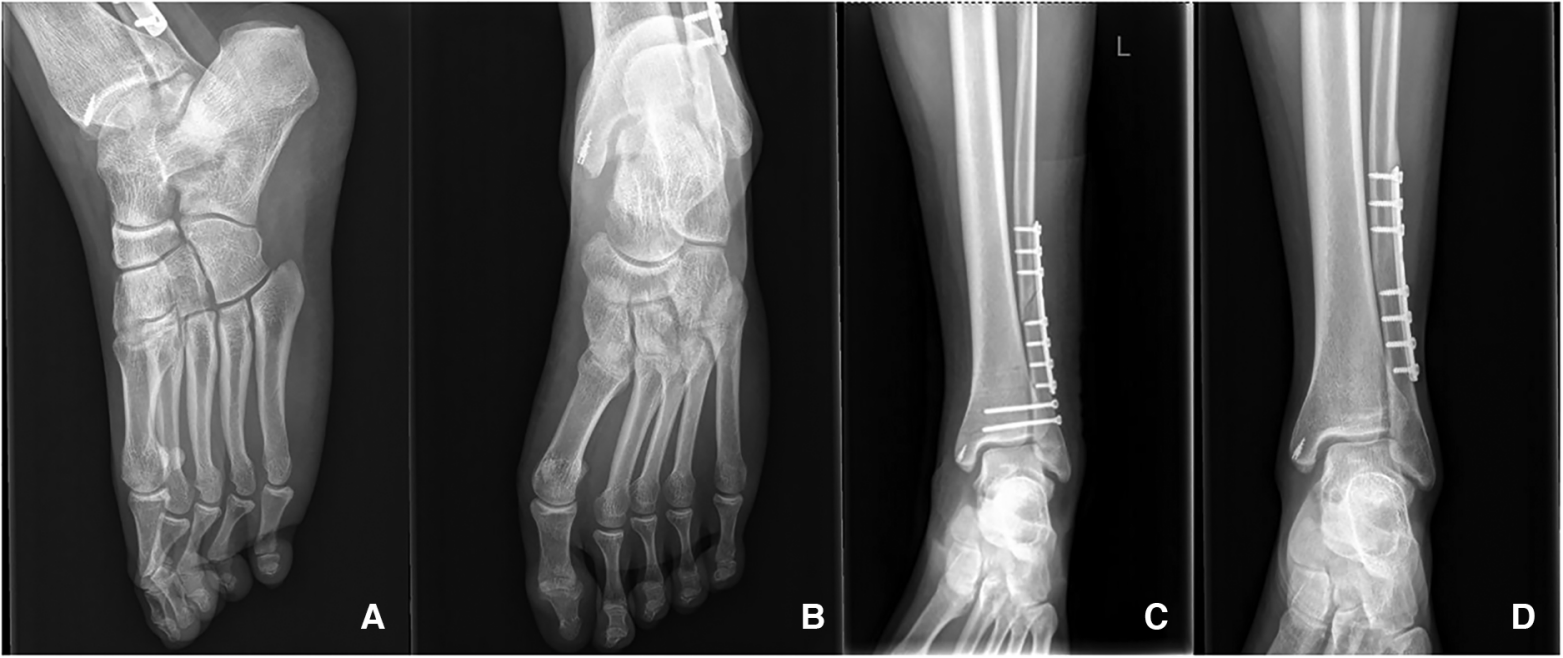
X-ray of the left foot and ankle 32 months after the primary operation. No dislocation of the IP and MP joints (**A,B**) and synostosis formation at the fracture site or interosseous membrane (**D**) was observed. Syndesmotic screws (**C**) were removed at postoperative 10 weeks after primary operation.

**Figure 3:**
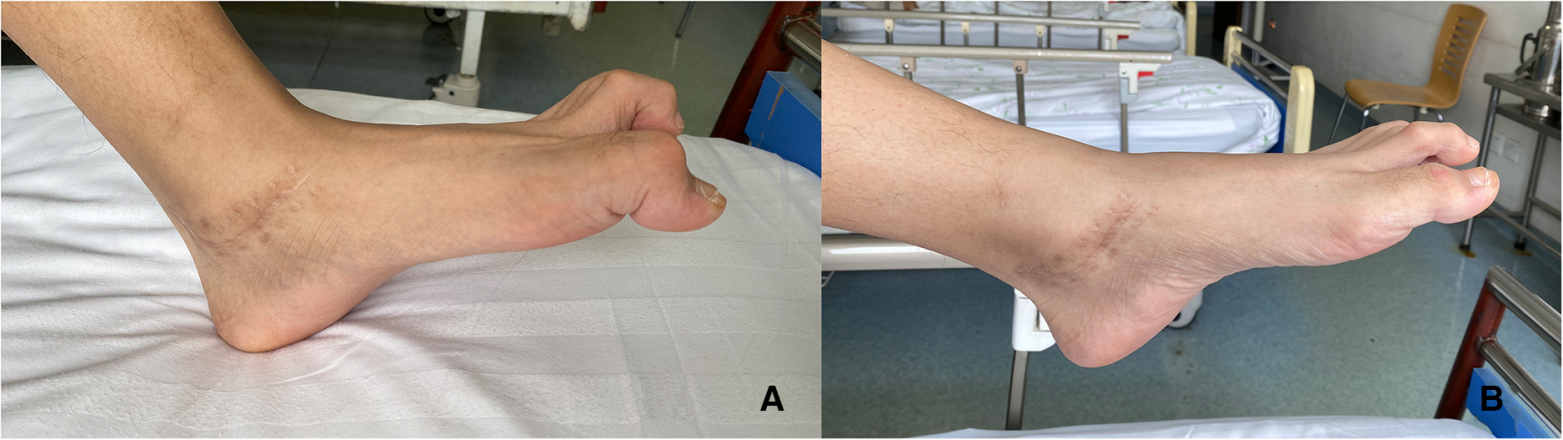
Macroscopic appearance of the checkrein deformity of the left hallux and second toe 32 months after the primary operation. (**A**) The deformity was exaggerated with ankle dorsiflexion. (**B**) The deformity was ameliorated with ankle plantar flexion.

**Figure 4:**
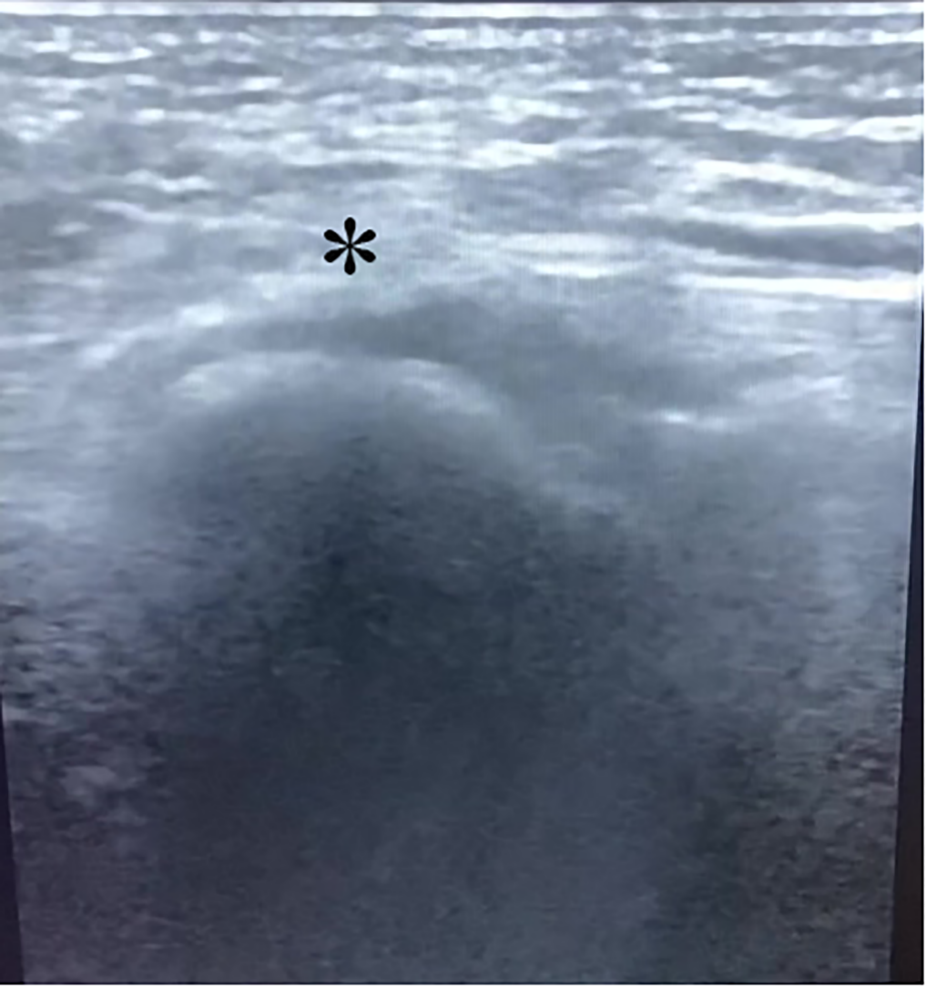
The asterisk indicated the presence of FHL tendon in the malleolar canal by the dynamic ultrasound.

**Figure 5:**
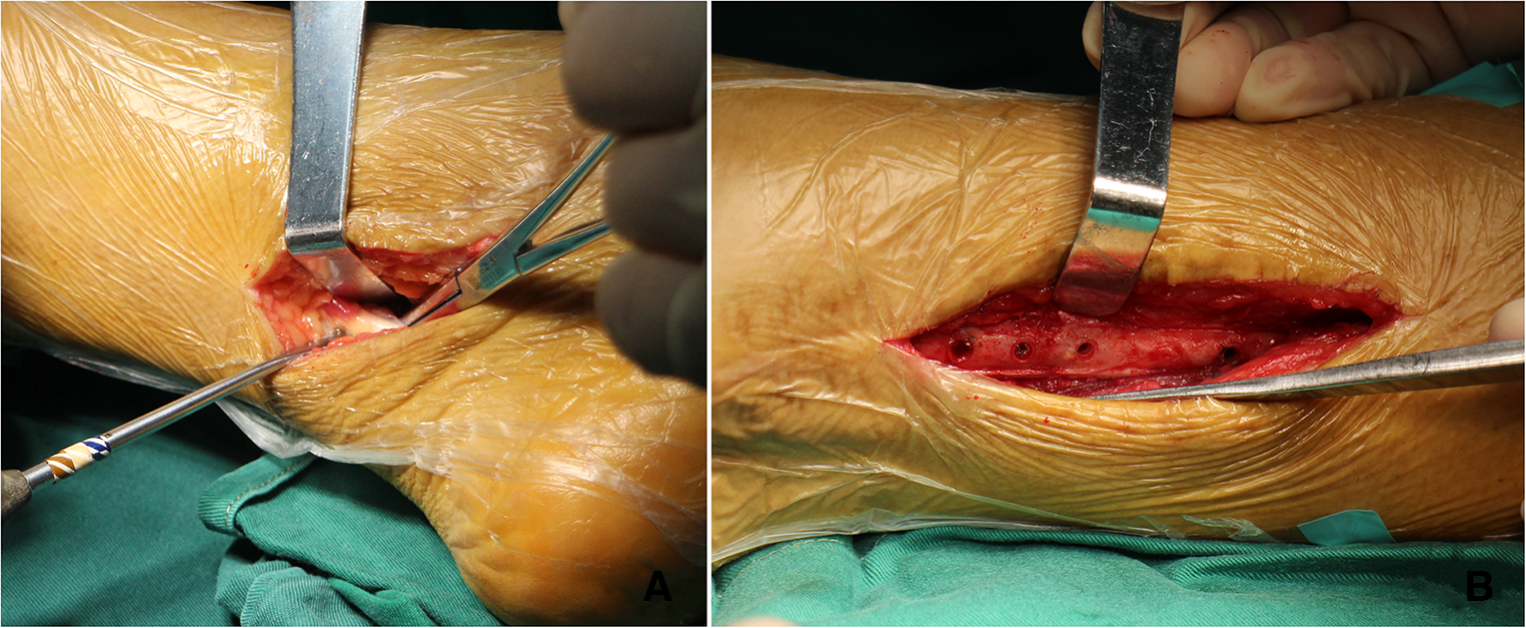
Incisions of the exploration operation and FHL tenolysis. (**A**) The glide of the FHL tendon was observed within the malleolar canal through posteromedial incision. (**B**) The FHL muscle belly was attached to the fibula in the deep posterior compartment.

**Figure 6:**
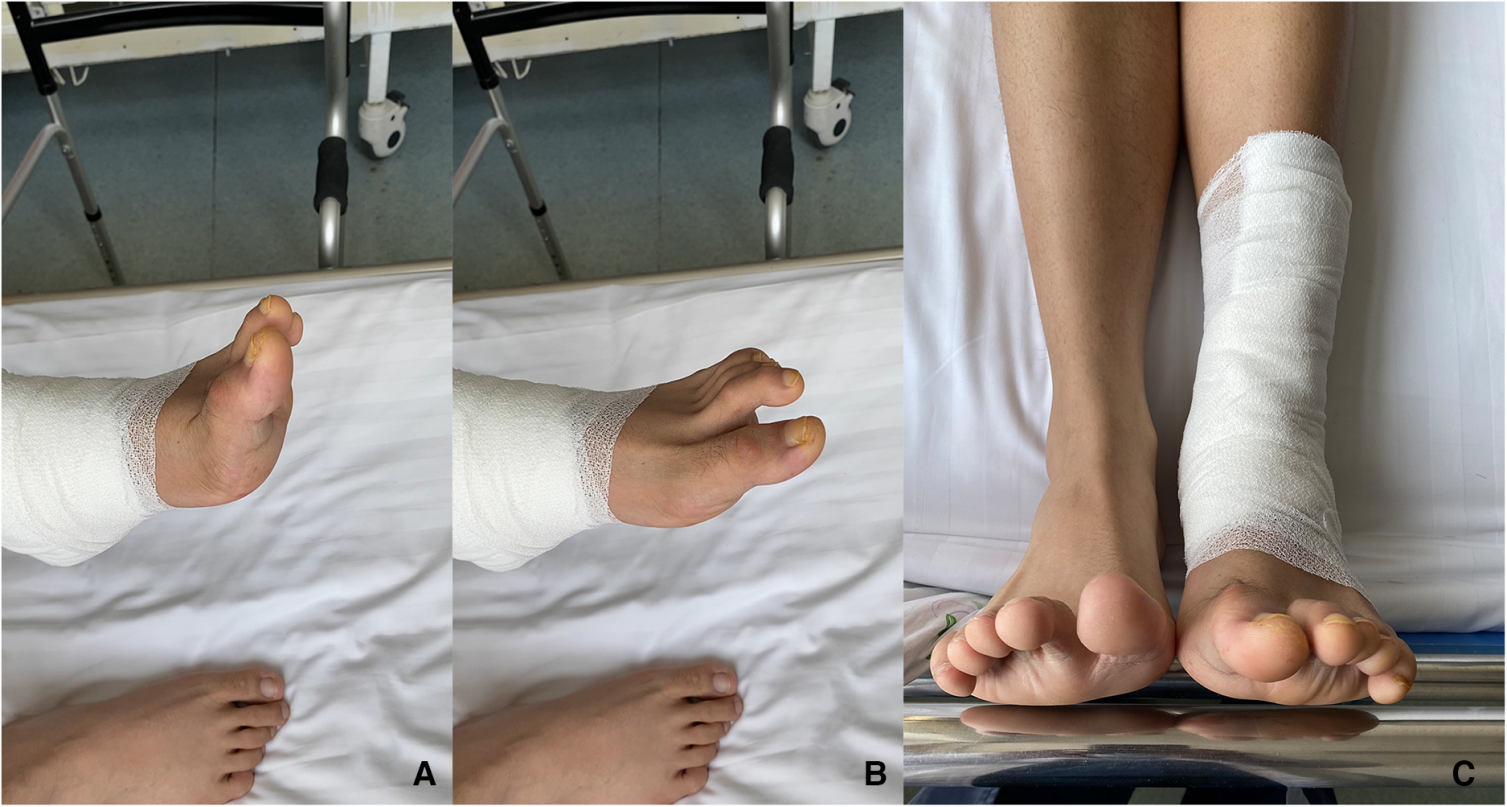
Macroscopic appearance of the left foot after surgical correction. Independent of the ankle position, dorsiflexion (**A**), plantar flexion (**B**), neutral (**C**), and the range of motion of the hallux and second toe was restored.

A 20-year-old male patient [98 kg, 181 cm, body mass index (BMI) = 29.9] was first admitted to our department with diagnosis of Lauge–Hansen PER stage IV malleolar fracture (Figure 1). ORIF with plates and screws was performed to reconstruct the stability of the left ankle joint. The syndesmotic screws were removed at 10 weeks postoperatively (Figure 2). In the follow-up, progressive clawing of the hallux and second toe of the left foot was observed at 3 months postoperatively; because of the lack of an evident effect on gait, the patient did not seek any medical help. The patient sought hardware removal and correction of claw toes 32 months after the primary operation. The physical examination revealed that the deformity of the hallux and second toe was exaggerated with ankle dorsiflexion and ameliorated with ankle plantar flexion (Figure 3). X-rays revealed no dislocation of the IP and MP joints. The fibular fracture healed with no synostosis formation at the fracture site or interosseous membrane (Figure 2). Dynamic ultrasonography indicated the presence of an excursion of the FHL tendon in the malleolar canal (Figure 4). Then, open exploration was performed through the former posteromedial and lateral approaches. First, the original posteromedial incision was used to expose the malleolar canal. After dissecting and protecting the neurovascular bundle, the glide of the FHL tendon was observed within the malleolar canal with ankle and foot motion through posteromedial incision (Figure 5). The retraction of the FHL tendon in the canal resulted in IP joint flexion but limited glide of the proximal musculotendinous unit. Second, original longitudinal fibular incision was used to remove the fibular hardware and explore the FHL muscle belly. After removing the fibular hardware, the FHL muscle belly was identified as being attached to the fibula with fibrous adhesion in the deep posterior compartment (Figure 5). Meanwhile, thorough tenolysis of the FHL muscle belly with fibrous adhesion release was performed. Retraction of the FHL tendon in the retromalleolar canal resulted in normal excursion of the FHL musculotendinous unit. At this stage, full extension of the hallux and second toe had been achieved. Immediate postoperative active motion exercises were initiated for the left foot and ankle. The patient was encouraged to walk in a weight-bearing manner with no immobilization of the lower extremities. Independent of the ankle position, the range of motion of the hallux was restored, and the checkrein deformity was corrected (Figure 6). In the 4-month follow-up, recurrence of the checkrein deformity was not observed.

## Discussion

In contrast to fixed claw toes caused by the IP joint lesions, checkrein deformity may be exaggerated or ameliorated by ankle and foot activity. This rare complication might be caused by intrinsic muscle dysfunction, tendon entrapment, or tethering induced by fracture callous formation or Volkmann contracture (3). It could be secondary to tibial, fibular, calcaneus, or talar fractures or dislocation of the ankle joint. Meanwhile, tethering or entrapment of the FHL tendon was the most typical cause (4–8). After a comprehensive literature review, posttraumatic checkrein deformity of the hallux has rarely been reported following Lauge–Hansen pronation external rotation malleolar fracture (6, 9–11). Malunited fracture (5), displaced hardware (12), and synostosis at the fracture site may result in limitations of FHL excursion. Another major identified cause was ischemic Volkmann contracture in the deep posterior compartment, which was occult and more likely to be ignored (13, 14). In this scenario, the potential for active flexion of the IP joint was irreparably lost.

Anatomically, the FHL originates from the posterior surface of the fibula in the deep posterior compartment, passes through the medial retromalleolar region, and inserts into the hallux. An invariable intertendinous connection is observed between the FHL and flexor digitorum longus (FDL) at the master knot of Henry (15).

Different surgical methods are available for correcting checkrein deformity, including FHL to extensor hallucis longus (EHL) transfer (16), lengthening the FHL with or without Z-plasty at the fracture site or midfoot (11, 17, 18), tenotomy at the forefoot level (5), or releasing tendon adhesion (19). Currently, the optimal surgical strategy is still not defined. Lengthening of the FHL tendon might be an option for the correction of checkrein deformity caused by diffuse ischemic contracture or poliomyelitis producing a fixed length phenomenon (19). Tenolysis is a feasible option for FHL tendon entrapment or tethering. All surgical plans should be tailored to the presumed cause of the checkrein deformity.

In contrast to the most common supination-external rotation type, the category of Lauge–Hansen PER stage IV appeared as medial mortise widening with deltoid ligament rupture, distal tibiofibular syndesmosis diastasis with interosseous membrane injury, fibular fracture above the syndesmosis, and posterior inferior tibiofibular ligament injury. Therefore, ORIF using a combination of medial and lateral approaches was usually performed in the primary operation with the aim of decreasing posttraumatic arthritis.

However, the checkrein deformity occurred 3 months postoperatively. In similar case reports, Leitschuh et al. (6) described flexion deformity of hallux secondary to Lauge–Hansen pronation-eversion stage IV injury. Due to the failure of the first operation, the patient underwent two surgical procedures at an interval of 8 months. The combination of the two operations might result in flexion contracture of the hallux. Adhesion release of the FHL corrected the deformity. Rosenberg and Sferra (9) described a hallux flexion deformity following a closed Salter-Harris type II ankle fracture caused by concomitant tethering of the FHL tendon combined with rupture of the posterior tibialis tendon (PTT). Tenolysis of the FHL and reconstruction of the PTT were performed to achieve normal motion of the hallux. Sanhudo and Lompa (10) and Lee et al. (11) introduced lengthening of the FHL tendon to correct the checkrein deformity following the malleolar fracture. The limitation in their studies was that the definite cause of the deformity was not clarified.

The soft tissue in patients with a Lauge–Hansen PER stage IV fracture might be damaged to varying degrees. Herein, we speculated that interosseous membrane injury and fibular fracture increased the risk of adhesion of the FHL muscle belly to adjacent tissues in our patient. Scarred fibroses following local hematoma might be an additional cause. Preoperative radiographic evaluation and ultrasonography provided useful information to confirm FHL adhesion. A possible adhesion site was located at the fibula in the deep posterior compartment. In addition, the tendinous interconnections between the FHL and FDL tendons might account for the deformity of both the hallux and the second toe. Therefore, sole tenolysis of the FHL sufficed to correct the deformity and improve the motion of the IP and MP joints of both the hallux and second toe with no need to explore FDL. Lengthening of the tendon was unnecessary for FHL adhesion and might even result in compromised foot function.

## Summary

This patient suffered from posttraumatic adhesion of the FHL in the deep posterior compartment, resulting in a checkrein deformity of the hallux and second toe. A thorough evaluation of the history, physical examination, ultrasonography, and radiographic evaluation could provide detailed information to confirm the pathology of FHL. The sole tenolysis of the FHL sufficed to correct the deformity of the hallux and second toe due to the intertendinous connection of the FHL and FDL.

## Data Availability

The original contributions presented in the study are included in the article/Supplementary Material, further inquiries can be directed to the corresponding author.
